# Discordant pathology following pancreatoduodenectomy for suspected intraductal papillary mucinous neoplasm: reassessment of cyst fluid genetic analysis

**DOI:** 10.1093/jscr/rjag798

**Published:** 2026-09-28

**Authors:** Ella Sibbersen, Dhruv J Patel, Hassam A Cheema, Ralph H Hruban, C Max Schmidt

**Affiliations:** Butler University, College of Liberal Arts and Sciences, Indianapolis, IN, United States; Department of Surgery, Biochemistry and Molecular Biology, Indiana University School of Medicine, Indianapolis, IN, United States; Department of Pathology, Indiana University School of Medicine, Indianapolis, IN, United States; The Sol Goldman Pancreatic Cancer Research Center, Department of Pathology, Johns Hopkins University School of Medicine, Baltimore, MD, United States; Department of Surgery, Biochemistry and Molecular Biology, Indiana University School of Medicine, Indianapolis, IN, United States

**Keywords:** pancreatic cysts, IPMN, molecular testing

## Abstract

A molecular analysis of pancreatic cyst fluid is revolutionizing the clinical management of pancreatic cysts. Yet, there remains need for ongoing correlation of molecular testing with surgical pathology. An ~70-year old male was found to have focal main pancreatic duct dilation up to 7 mm at the pancreatic head/neck on imaging without any discrete mass lesions. Duct fluid analysis found elevated carcinoembryonic antigen and decreased glucose level suggestive of intraductal papillary mucinous neoplasm (IPMN). Yet, molecular testing revealed no mutations. In the absence of a mass lesion, he was recommended surveillance. A year later, he had stable imaging and underwent repeat molecular testing, which revealed *KRAS* and *GNAS* mutations. He underwent pancreatoduodenectomy for suspected main duct IPMN. Final pathology revealed low-grade, multifocal pancreatic intraepithelial neoplasia, not IPMN. Ongoing assessment of concordance between molecular testing and postoperative pathology after pancreatectomy is needed to refine the multimodal diagnostic evaluation of pancreatic cysts.

## Introduction

Intraductal papillary mucinous neoplasm (IPMN) is a cystic lesion of the pancreas with potential to progress to invasive carcinoma. Pancreatic cysts are present in 2.5% of the population, with half of cysts being mucin-producing (IPMN or mucinous cystic neoplasm) precursor lesions to pancreatic ductal adenocarcinoma (PDAC) [1, 2]. IPMNs are often asymptomatic. A multimodal approach is required to both diagnose and assess the malignancy potential of IPMN. Symptoms/signs, radiographic features, cyst fluid cytology, and molecular analyses all help stratify IPMN malignancy risk [3–5].

Molecular analysis of pancreatic cyst fluid has been examined prospectively and has correlated *GNAS* mutations with 90% sensitivity and 100% specificity for mucin-producing cysts [1, 6]. Compared to standard clinical, imaging, cytopathologic, and cyst fluid biomarker analysis, the addition of molecular analyses has demonstrated superior performance in the diagnosis of cystic neoplasms [7]. Additionally, molecular testing provides value in the stratification of which mucin-producing cystic lesions may develop advanced neoplasia, further enhancing operative decision-making. Yet, the limited operative cases included for validation remains one limitation of the literature supporting the utility of molecular analysis for pancreatic cysts. A patient with suspected main duct involved IPMN based on molecular testing, but who had pancreatic intraepithelial neoplasia (PanIN) with no evidence of IPMN on final pathology is herein presented.

## Case report

The patient was an ~70-year old male who presented to another hospital with acute onset of nausea, vomiting, and diarrheoa in January 2024. The symptoms were attributed to viral gastroenteritis; however, cross-sectional imaging of the abdomen was notable for a dilated main pancreatic duct (MPD), measuring up to 7 mm at the head/neck region, with no discrete lesions. Serum tumour markers [carcinoembryonic antigen (CEA) and carbohydrate antigen (CA) 19–9] obtained at gastroenterology follow-up were within normal limits. The patient had no significant risk factors for pancreatic cancer. He underwent an endoscopic ultrasound with fine-needle aspiration (EUS-FNA) of the MPD in April 2024. Duct fluid analysis was notable for a CEA of 677 ng/ml (reference: >192 ng/ml concerning for mucinous etiology) and a glucose of 26 mg/dl (reference: ≤50 mg/dl concerning for a mucin-producing cyst). Molecular testing revealed no mutations. The patient was referred to the Indiana University Health Pancreatic Cyst and Cancer Early Detection Clinic (PCC-EDC).

Despite radiographic and biochemical data which suggested a main duct IPMN, it remained unclear whether the patient had IPMN due to lack of a discrete lesion. Upon evaluation in June 2024, continued surveillance was offered over surgical resection. Follow-up MRI in September 2024 and March 2025 revealed stable MPD dilation. The patient remained asymptomatic. Repeat EUS-FNA was performed in April 2025 with molecular testing revealing both *KRAS* {p.G12V c.35G > T [Allelic Frequency (AF) = 14%], p.G12R c.34G > C [AF = 5%], and p.G12D c.35G > A [AF = 2%]} and *GNAS* (pR201H c.602G > A [AF = 1%]) mutations. He was reassessed in PCC-EDC and counseled that his risk of cancer development from suspected IPMN malignant progression was likely greater than the surgical morbidity/mortality of pancreatoduodenectomy. The patient requested additional time to consider his options. He was seen in clinic in August 2025 with stable labs and imaging (Fig. 1). He then elected for operative management.

**Figure 1:**
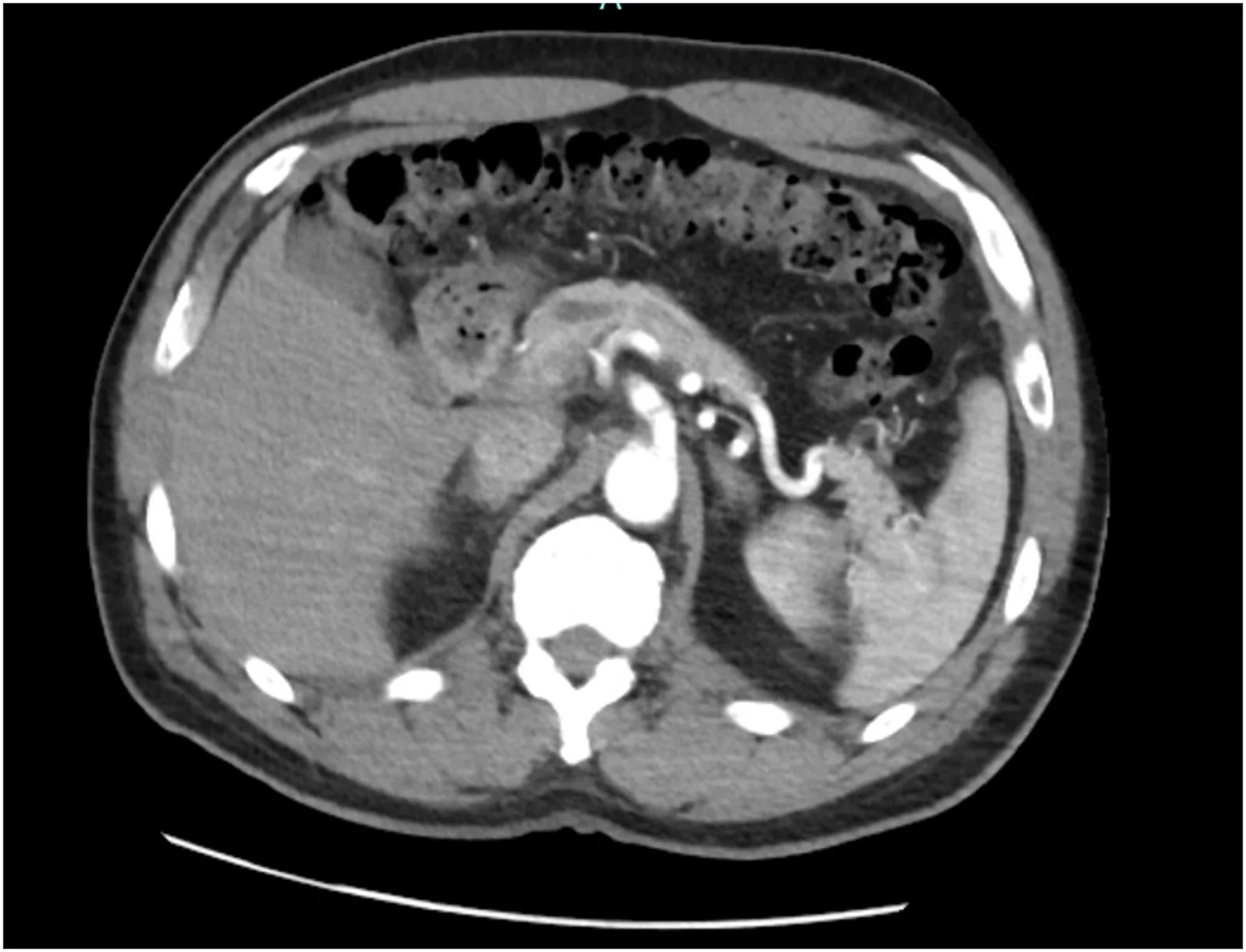
Computed tomography imaging of the patient’s main pancreatic duct dilation five days prior to surgery.

He underwent a pylorus-preserving pancreatoduodenectomy. His intraoperative course was uneventful. Frozen sections from the pancreatic neck and bile duct underwent pathological review and were negative for high-grade dysplasia or invasive cancer. He developed a grade B postoperative pancreatic fistula (POPF) with associated delayed gastric emptying and non-occlusive thrombus of the superior mesenteric vein. The POPF was managed conservatively with prolonged surgical drainage, octreotide, parenteral nutrition, and intravenous antibiotics.

Final pathology revealed the head of the pancreas to have low-grade, multifocal PanIN, with no evidence of IPMN (Fig. 2). Two independent pathologists evaluated the patient’s pathology, both agreeing with the pathologic assessment of PanIN.

**Figure 2:**
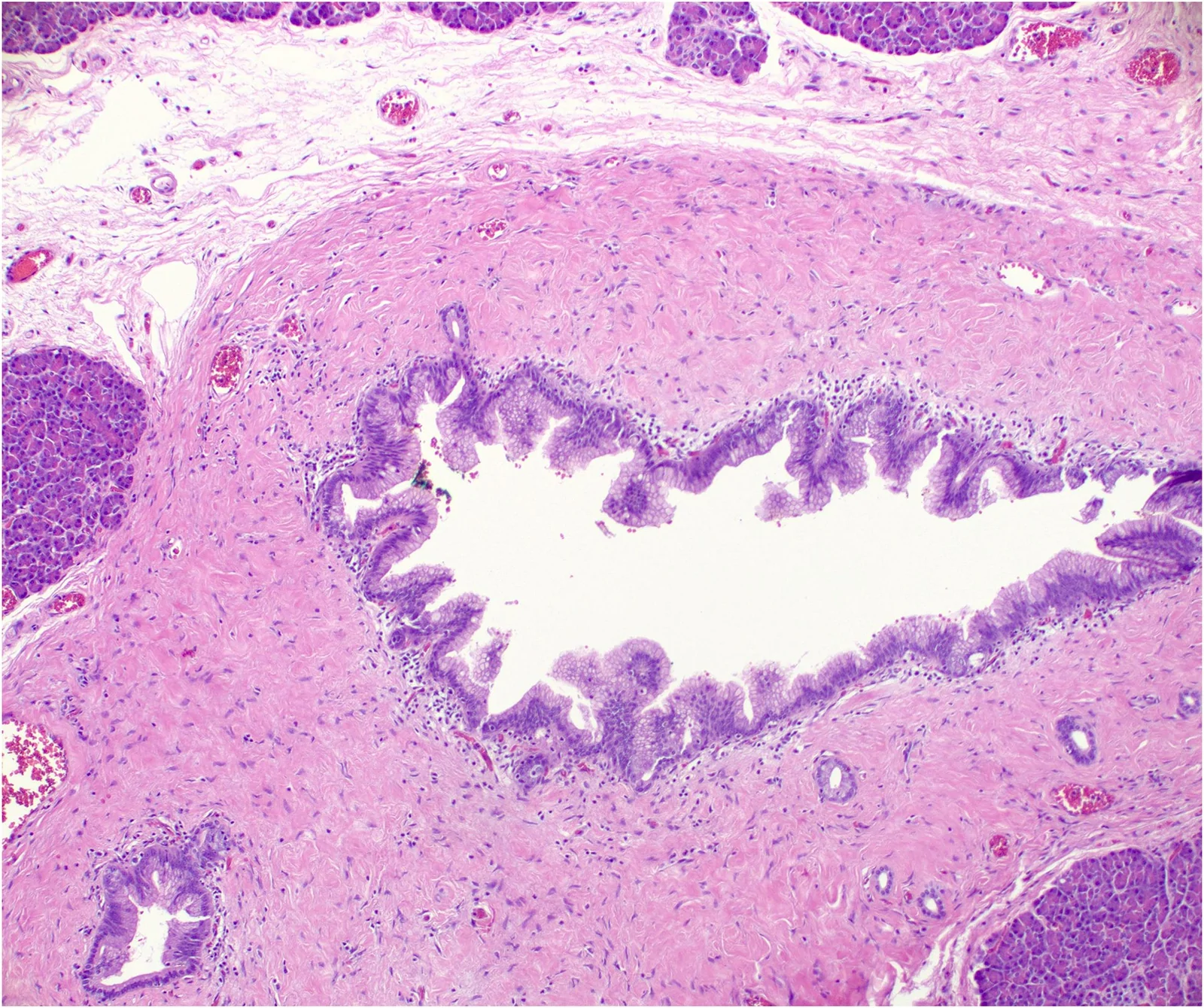
Histologic section of the pancreatoduodenectomy specimen with main pancreatic duct demonstrating low-grade pancreatic intraepithelial neoplasia (PanIN).

## Discussion

Although our patient had isolated MPD dilation with duct fluid analysis revealing *KRAS* and *GNAS* mutations, he did not have IPMN on final postoperative pathology, highlighting the significance of multimodal diagnostic assessment of suspected cystic lesions of the pancreas. Several elements of this case warrant further discussion.

The patient was suspected as having IPMN based on a multimodal evaluation, including radiographic (dilated MPD), biochemical (elevated CEA, low glucose) and molecular testing (*GNAS* and *KRAS* mutations). It has been reported that *GNAS* mutations are commonly found in mucin-producing cystic lesions, present in up to 66% of IPMN [8]. In contrast, a review of 77 PanIN cases found a 3% incidence of *GNAS* mutations [5]. *KRAS* is among the most important regulatory genes in pancreatic neoplasms and exhibits high mutation rates in both IPMN and PanIN [9, 10]. Yet, there is limited literature linking the presence of both mutations in IPMN. A prior analysis of 68 resected cystic lesions found 5 cases with mutations of both *KRAS* and *GNAS*, all of which were IPMNs [11]. The present case was notable for *GNAS* and *KRAS* mutations found on preoperative molecular testing in a patient with only PanIN on final surgical pathology.

By diagnostic criteria, IPMNs must be >1 cm in diameter, whereas PanIN lesions are microscopic and usually <0.5 cm in diameter [12]. IPMNs often extend from larger ducts to smaller, and PanIN lesions typically arise in smaller ducts but have the capacity to involve larger ones [13, 14]. Despite general criteria for distinguishing PanIN from IPMN, histologic distinction is not always possible. Incipient IPMN is a separate term for cystic lesions between IPMN and PanIN [11, 12]. Incipient IPMNs are <1 cm in diameter but have similar epithelial features as IPMNs (long papillae, prominent mucin production) [12]. Pathologic assessment of the current case was definitive for PanIN as the lesion lacked longer papillae.

This case highlights the challenges of molecular testing in the diagnostic evaluation of pancreatic lesions. Future directions involve assessing how results of molecular testing platforms change over time and evaluating concordance between molecular testing and postoperative pathology after pancreatectomy in larger series.
